# A Pitfall in Transrectal Prostate Biopsy: Malakoplakia Evaluation of Two Cases Based on the Literature Review

**DOI:** 10.1155/2014/150972

**Published:** 2014-04-29

**Authors:** Dudu Solakoglu Kahraman, Sevil Sayhan, Gulden Diniz, Duygu Ayaz, Tugba Karadeniz, Ertan Can

**Affiliations:** ^1^Pathology Department, Tepecik Training and Research Hospital, Izmir, Turkey; ^2^Urology Department, Tepecik Training and Research Hospital, Izmir, Turkey

## Abstract

Malakoplakia is a rarely seen inflammatory condition that is considered to develop secondary to a chronic *Escherichia coli* infection. Although malakoplakia usually affects the genitourinary tract, it may also be observed in the colon, stomach, lungs, liver, bones, uterus, and skin. Malakoplakia of the genitourinary system usually involves the bladder, whereas it may also affect the prostate along with the bladder. Malakoplakia of the prostate is very rare, and it may be clinically mistaken for prostatic malignancies. Definitive diagnosis is only possible through histopathological examination. This study elaborates on two patients who presented to our hospital in 2013 with high PSA levels. The primary clinical consideration was prostate carcinoma. However, these two cases were diagnosed as malakoplakia based on the results of histopathological analysis of the transrectal prostate biopsy specimen.

## 1. Introduction

Malakoplakia is a rare granulomatous inflammatory condition, which usually involves the urinary bladder, and it is considered to develop as a result of a defective immune response to bacterial agents [1, 2]. Lesions in the genitourinary system present as papules, plaques, and ulcerations [1]. In the histopathological examination, malakoplakia is characterized by the presence of foamy histiocytes with distinctive basophilic inclusions, which are known as Michaelis-Gutmann bodies (MGBs). Malakoplakia of the genitourinary system is more common in women than in men. It is usually observed between the fifth and seventh decades of life. In male patients, malakoplakia may also involve the prostatic tissue along with the bladder [1]. However, very rarely malakoplakia of the prostate does not involve the bladder. [1–3]. It is usually mistaken for malignancy, as it leads to a formation of a prostatic mass and thickening of the bladder wall [2]. This study presents two cases with a primary clinical consideration of malignancy, which were later diagnosed as malakoplakia of the prostate based on the histopathological examination of prostate needle biopsy results.

**Case 1**

**Figure 1:**
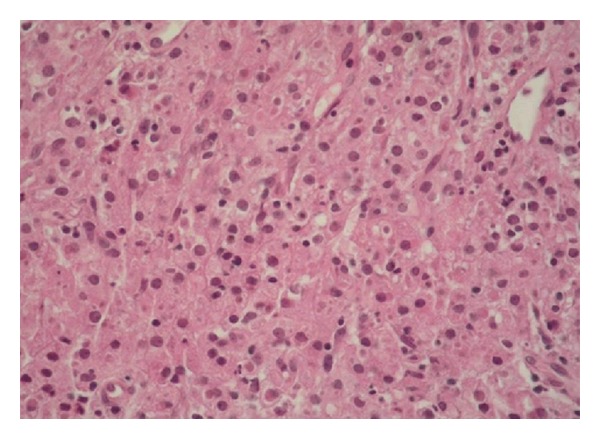
Note the diffuse eosinophilic histiocytes infiltration in prostate tissue and presence of intracytoplasmic basophilic inclusions (HE ×200).

**Figure 2:**
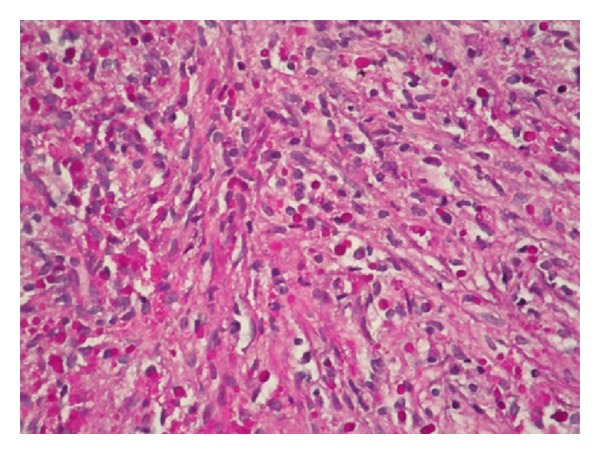
PAS positive Michaelis Gutmann bodies (PAS ×200).

A 70-year-old male patient presented to our hospital with complaints of burning and discomfort during urination. The patient's history was unremarkable. Prostatic enlargement and nodules were detected during his prostate examination. His serum PSA level was 14.1 ng/mL. Urinalysis revealed the presence of bacteriuria.* Escherichia coli sp. *was grown in his urine culture. Although cystoscopy did not yield any specific results, his pelvic ultrasonography revealed a very large prostatic hypertrophy. The patient underwent transrectal ultrasound-guided 14 quadrant needle biopsy of the prostate with the primary clinical consideration of malignancy. Histopathological examination demonstrated diffuse infiltration of eosinophilic histiocytes (von Hansemann cells), plasma cells, and lymphocytes within fibromuscular stroma of the prostate. Histiocytes had distinctive intracytoplasmic inclusions with targetoid appearance (MGB) (Figure 1). PAS staining revealed the presence of MGBs (Figure 2).

**Figure 3:**
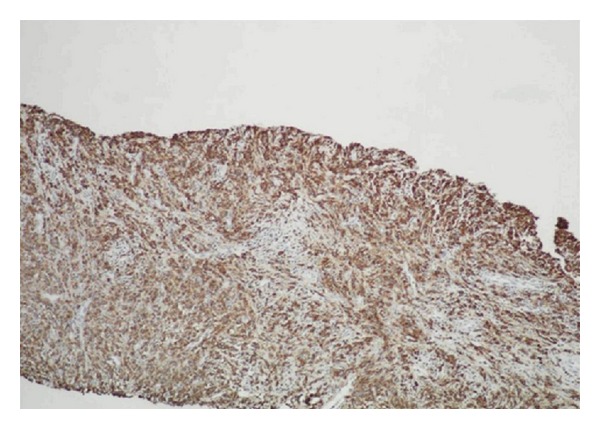
Immunohistochemically detected diffuse histiocytic infiltration with anti-CD68 antibody (DAB ×100).

Immunohistochemical staining demonstrated diffuse positivity and strong immune reactivity for CD68 (Figure 3). Levels of high molecular weight cytokeratin (HMWCK), prostate-specific antigen (PSA), and prostate-specific acid phosphatase (PSAP) were within normal limits. The case was diagnosed as malakoplakia based on the above mentioned results. One month after receiving antibiotherapy, the patient underwent open prostatectomy. The histopathological examination of prostatectomy specimen disclosed findings of adenomatous hyperplasia of the prostate and chronic prostatitis. None of the findings suggested malakoplakia.

**Case 2**

A 69-year-old male patient presented to our urology outpatient clinic with complaints of burning and discomfort during urination. Urinalysis revealed the presence of bacteriuria.* Escherichia coli sp. *was grown in his urine culture. His serum PSA level was 100 ng/mL. Pelvic ultrasonography revealed nothing but an increased prostate volume and parenchymal calcification. The patient underwent 6-quadrant transrectal needle biopsy of the prostate with the primary consideration of malignancy. Histopathological examination of the biopsy material indicated diffuse infiltration of von Hansemann histiocytes into prostatic tissues. Like in Case  1, immunohistochemical staining revealed strong immune reactivity of these cells for CD68. Levels of pancytokeratin, alpha-methylacyl-CoA racemase (AMACR), and leucocyte common antigen (LCA) were within normal limits. Like in Case  1, PAS histochemical staining revealed intracytoplasmic bodies (Michaelis-Gutmann bodies) (MGBs). The case was diagnosed as malakoplakia. Since suspicion for malignancy could not be ruled out, thoracic and lower abdominal CT, and whole-body scans were obtained without any evidence of malignancy. Neither of the patients had bladder lesions.

## 2. Discussion

Michaelis and Gutmann were the first to define malakoplakia in 1902. In 1903, Hansemann coined the term malakoplakia, which was derived from a combination of malakos (soft) and plakos (plaque) in ancient Greek [4, 5]. Malakoplakia of the prostate was defined for the first time by Carruthers in 1959. Malakoplakia is a rare inflammatory condition, which usually involves the genitourinary system, manifesting yellow soft plaques or nodules characterized by accumulation of macrophages. Definitive diagnosis requires histopathological examination. Microscopically, malakoplakia is characterized by the presence of von Hansemann macrophages including MGBs, and it is observed in immunocompromised patients with recurrent* E. coli* infections of the urinary system [4, 6, 7].

Malakoplakia is a rarely seen infection, and in 80 to 90% of patients' urine cultures predominately* E. coli* and* Klebsiella pneumoniae *grow [8]. It is considered that a defective immune response to microbial agents is responsible pathogenetic mechanism [2–4]. Some authors reported the presence of an association between the disease and immunosuppression [1, 3]. MGBs are thought to consist of phagolysosomes including bacterial debris. MGBs were found to contain calcium hydroxyapatite and iron. Therefore, PAS, Prussian Blue, and von Kossa dyes are used for histochemical staining [2, 7, 8]. Malakoplakia of the prostate may be observed with or without bladder involvement. Cases of prostatic malakoplakia without bladder involvement are very rare. Bladder ultrasonography did not reveal any specific abnormality in our two cases. They were considered as cases of malakoplakia with prostatic involvement. Malakoplakia of the prostate may accompany a tumour or it may rarely be observed in isolation.* Escherichia coli* propagation was observed in urine cultures of both cases. Malakoplakia may be mistaken for nonspecific granulomatous prostatitis in the histopathological analysis of the prostate needle biopsy specimen. Cases that morphologically resemble malakoplakia but do not contain MGBs are referred to as the nodular histiocytic prostatitis. Intravesical* Bacillus* Calmette-Guérin (BCG) therapy for bladder carcinoma may cause granulomatous prostatitis [1, 6]. Our cases did not have bladder tumour history. As was the case for us, malakoplakia of the prostate may be mistaken for prostatic carcinoma since it leads to the development of hard nodules that may be clinically confused with carcinoma during digital rectal examinations for bladder obstruction and prostate. Misdiagnosis may occur especially in the microscopic examination of transrectal needle biopsy material, since the histiocytic infiltration observed in malakoplakia resembles the tumour cells in clear-cell prostate carcinoma [7]. Thus, a careful histopathological examination should accompany histochemical and immunohistochemical tests during the diagnostic workup for malakoplakia.

Malakoplakia of the lower genitourinary system consists of sharply bordered lesions usually without pelvic organ invasion.

Treatment alternatives include antibiotherapy or surgery, depending on the localisation of the involvement and degree of invasion [4]. Antibiotics penetrate into the macrophage cell membrane and cure the bacterial infection. In trimethoprim sulfamethoxazole therapy, trimethoprim kills the undigested bacteria in the malakoplakia macrophages. Sulfamethoxazole penetrates into the macrophage and proves useful for patients with advanced malakoplakia. In addition to these agents, bethanechol improves phagocytic bactericidal activity by increasing the cGMP level. Open surgical resection or TUR-P is performed, if conservative treatment proves to be insufficient for the treatment of malakoplakia [3, 4, 7, 9].

In Case  1, one month after receiving antibiotherapy, the patient underwent open prostatectomy, whereas the patient in Case  2 received antibiotherapy only. Both patients had not demonstrated any pathological findings in routine controls.

This study discussed the above-mentioned two cases in light of the literature in that they were rarely seen and clinically mistaken for malignancy. These cases can also be considered as pitfalls for the pathologist since they could be mistaken for malignancy.
